# Risk Estimation of Severe Primary Graft Dysfunction in Heart Transplant Recipients Using a Smartphone

**DOI:** 10.31083/RCM25170

**Published:** 2025-01-08

**Authors:** Souhila Ait-Tigrine, Roger Hullin, Elsa Hoti, Matthias Kirsch, Piergiorgio Tozzi

**Affiliations:** ^1^Internal Medicine, Lausanne University Hospital CHUV Lausanne, 1011 Lausanne, Switzerland; ^2^Cardiology, Lausanne University Hospital CHUV Lausanne, 1011 Lausanne, Switzerland; ^3^Lausanne University School of Medicine, 1005 Lausanne, Switzerland; ^4^Cardiac Surgery, Lausanne University Hospital CHUV Lausanne, 1011 Lausanne, Switzerland

**Keywords:** heart transplant, heart transplantation, primary graft dysfunction, primary graft failure, temporary circulatory support, ECMO, risk prediction score

## Abstract

**Background:**

Currently, there are no standardized guidelines for graft allocation in heart transplants (HTxs), particularly when considering organs from marginal donors and donors after cardiocirculatory arrest. This complexity highlights the need for an effective risk analysis tool for primary graft dysfunction (PGD), a severe complication in HTx. Existing score systems for predicting PGD lack superior predictive capability and are often too complex for routine clinical use. This study sought to develop a user-friendly score integrating variables from these systems to enhance the efficacy of the organ allocation process.

**Methods:**

Severe PGD was defined as the need for mechanical circulatory support and/or death from an unknown etiology within the first 24 hours following HTx. We used a meta-analytical approach to create a derivation cohort to identify risk factors. We then applied a logistic regression analysis to generate an equation predicting severe PGD risk. We used our previous experience in HTx to create a validation cohort. Subsequently, we implemented the formula in a smartphone application.

**Results:**

The meta-analysis comprising six studies revealed a 10.5% ( 95% confidence interval (CI): 5.3–12.4) incidence rate of severe PGD and related 30-day mortality of 38.6%. Eleven risk factors were identified: female donors, female donor to male recipient, undersized donor, donor age, recipient on ventricular assist device support, recipient on amiodarone treatment, recipient with diabetes and renal dysfunction, re-sternotomy, graft ischemic time, and bypass time. An equation to predict the risk, including the 11 parameters (GREF-11), was created using logistic regression models and validated based on our experience involving 116 patients. In our series, 29 recipients (25%) required extracorporeal membrane oxygenation support within 24 hours post-HTx. The overall 30-day mortality was 4.3%, 3.4%, and 6.8% in the non-PGD and severe PGD groups, respectively. The area under the receiver operating characteristic (AU-ROC) curve of the model in the validation cohort was 0.804.

**Conclusions:**

The GREF-11 application should offer HTx teams several benefits, including standardized risk assessment and bedside clinical decision support, thereby helping minimize the risk of severe PGD post-HTx.

## 1. Introduction

Heart transplantation (HTx) remains the therapeutic modality closest to a cure for eligible patients with end-stage heart failure despite the recent progress made regarding long-term mechanical circulatory support therapies [1, 2]. Despite the increased medical complexity of transplant recipients, long-term HTx results have significantly improved over the last 20 years, with the 5-year mortality rate reduced by half [1]. Yet, at the same time, the 30-day mortality rate after HTx remains unchanged, ranging from 5% to 10% [2].

Excepting rare surgery-related complications and fulminant infections, the inability of the transplanted heart to generate adequate cardiac output in the early post-surgery phase is the primary determinant of early post-transplant mortality. Primary graft dysfunction (PGD) comprises all dysfunctions of unknown origin, after excluding hyperacute rejection, severe pulmonary hypertension, or recognized intraoperative complications [2]. PGD accounts for at least two-thirds of early graft dysfunctions, with the diagnosis usually made within 24 hours (h) post-transplantation [3, 4].

In the literature, the incidence of this devastating complication varies from 2% to 28%. Thus, attention must be paid to the organ allocation process to prevent PGD, although its etiology remains unclear [5, 6, 7, 8, 9]. PGD occurrence is associated with specific parameters of donor, recipient, and surgical procedures that are both heterogeneous and difficult to weigh up during organ allocation.

Existing scoring systems are complex and challenging to use in routine practice. Furthermore, these systems also vary in parameters, leading to inconsistent risk assessments, and often do not account for recent technological advancements, limiting their relevance in current practices. Therefore, there is a need for a more straightforward and effective tool. Our study sought to fill this gap by developing and validating a more comprehensive scoring system. We conducted a meta-analysis to identify key risk factors and integrated them into a user-friendly model. We hypothesized that our scoring system would provide better predictive accuracy and ease of use, improving decision-making in HTxs.

## 2. Materials and Methods 

### 2.1 Study Primary Endpoint 

The study primarily sought to identify risk factors, also identified as variables, for severe PGD within the first 24 h following HTx, thus requiring prolonged mechanical circulatory support or causing the patient’s death. The variables were related to the donor, recipient, and surgical procedure.

### 2.2 Data Sources and Searches

PGD risk prediction equation development started with the derivation cohort construction based on a meta-analysis of literature data. To identify relevant studies that have investigated risk factors associated with PGD after HTx, we systematically searched MEDLINE, EMBASE, and Cochrane databases of systematic reviews using the following keywords: “heart transplant”, “heart transplantation”, “primary graft dysfunction”, “primary graft failure”, “temporary circulatory support”, and “extracorporeal membrane oxygenation (ECMO)”.

### 2.3 Study Eligibility Criteria

We included studies that enrolled adult HTx recipients, evaluated any factor associated with PGD using multivariable analysis (Cox proportional hazards models, logistic regression models), and reported at least 10 severe PGDs. To focus on the contemporary definition of PGD, we excluded studies published before 2010 and considered those published until October 2022. We did not restrict study selection by design or publication type if they provided sufficient information to generate effect estimates for any predictor. If two studies assessed the same population and predictors, we included the study with the largest sample size. Two reviewers independently screened all titles and abstracts using a standardized study eligibility form (see **Supplementary A** in the **Supplementary Material**). For studies deemed potentially eligible, either of the reviewers evaluated the full text and then agreed on their inclusion.

### 2.4 Data Extraction and Quality Assessment

Three reviewers extracted data from eligible studies using a standardized form (see **Supplementary B** in the **Supplementary Material**). We collected data related to population characteristics, including donor and recipient age and sex, sex mismatch, predicted heart mass, graft total ischemic time and cardiopulmonary bypass time (Table 1), postoperative ECMO use, and 30-day survival. Reviewers also extracted data relevant to the definition of predictors, effect estimates, confidence intervals (CIs), and the definition of outcomes.

**Table 1.** **Variables were considered to identify independent risk factors for PGD**.

	Donor	Recipient	Surgical procedure
Variables	Age	Preoperative circulatory support	Graft ischemic time
	Gender	Age	CPB time
	Gender mismatch	Gender	Blood transfusions
	LV mass mismatch	Blood group	
	Blood group	Anemia	
	Cause of death	Diabetes	
	Left ventricular hypertrophy	Re-sternotomy	
	Inotropic requirement	Amiodarone treatment	
		Pulmonary artery resistance	

CPB, cardiopulmonary bypass; LV, left ventricle; PGD, primary graft dysfunction.

Study quality and risk of bias were assessed based on the Quality Prognostic Studies (QUIPS) [10] tool. This instrument considers study participant selection, loss to follow-up, prognostic factors, outcome measurements, confounding factors, and statistical analysis and reporting. If five or more domains presented a low risk of bias, the overall risk was low; otherwise, the risk of bias was deemed high [11].

### 2.5 Data Synthesis and Statistical Analysis

The research findings presented point estimates along with their corresponding 95% CIs using hazard ratio (HR), odds ratio (OR), or relative risk (RR). In studies with stratified groups showing a linear association between the predictor and the outcome, we averaged the beta coefficients across categories to obtain the effect estimate associated with a unit change for the meta-analysis. We performed a meta-analysis to combine the effect sizes from individual studies using the random-effects model and calculated the summary effect sizes and their corresponding CIs for each predictor variable. The meta-analysis was performed according to the Preferred Reporting Items for Systematic Reviews and Meta-Analyses (PRISMA 2020) statement [12]. Categorical data were expressed as absolute values and relative frequencies (%), and continuous variables were expressed as the mean and standard deviation (SD). Dichotomous variables were expressed as percentages. Baseline characteristics were compared using the Student’s *t*-test for continuous variables and the chi-square test for categorical variables. Non-normally distributed continuous variables were compared using non-parametric methods (Wilcoxon’s rank sum test). A value of *p* < 0.05 was considered statistically significant.

We identified the parameters in each study that were statistically significant in predicting PGD. More specifically, during data extraction, we first conducted an unadjusted analysis to assess the relevance of each variable. Variables that reached a *p*-value < 0.05 in this preliminary analysis were included in the subsequent multivariable analysis.

### 2.6 Logistic Regression Modelling

Python with scikit-learn was the software (version 3.10.6, 2022, located at https://python-fiddle.com/examples/sklearn) used for statistical analysis. Based on the meta-analysis results, we selected predictor variables that showed statistically significant associations with the outcome (*p* < 0.05). We fitted the logistic regression model using the prepared dataset, with severe PGD as the binary outcome variable and the selected predictor variables as independent variables. A Cox proportional hazards regression model was used to analyze the contributions of variables to the outcomes. For each variable, we considered the highest regression coefficient (RC) related to PGD and used it in the equation. Throughout the analysis, missing data were not imputed. Instead, we performed the study on a complete-case basis, meaning only cases with no missing data were included in the regression model [13]. This approach was chosen to ensure the accuracy and integrity of our findings by relying solely on fully available data. While this method reduces potential biases from imputation, it may limit sample size, which was acknowledged as a limitation in our study. The decision to use a complete-case analysis was based on the extent and nature of missing data, which we have deemed necessary to maintain the robustness of our results.

We calculated the intercept value and estimated the coefficients of the selected parameters based on a method called maximum likelihood estimation with iterative optimization techniques.

The formula for the score calculation was as follows:

intercept + (parameter 1 × coefficient parameter 1) + (parameter 2 × coefficient parameter 2) + …. = y

sigmoid(y) = 1/(1 + exp(-y))

we then expressed this value as a percentage, representing the estimation of the outcome risk in a given patient.

### 2.7 Model Validation

To determine the stratification method with the best discriminatory ability for PGD occurrence, we analyzed the area under the receiver operating characteristic (AU-ROC) curve or statistical C. This data analysis is considered a reliable method to distinguish patients evolving to severe PGD from those not changing when the AU-ROC curve is above 0.7 (1 corresponds to a method that can perfectly discriminate between PGD or not) [14].

### 2.8 Validation Cohort

Upon receiving ethical clearance from the Swiss Ethic Authority for Human Research, CER-VD 2022-00562, on September 6, 2022, we retrospectively collected data concerning all consecutive adult patients (≥18 years old) who underwent isolated orthotopic HTx in our institution between January 2013 and January 2022 to establish the validation cohort. Patients with combined organ transplants, donors after circulatory death (DCD), and patients for which it was not possible to retrieve all the information were excluded. In addition to the parameters illustrated in Table 1, we collected data on ECMO (venous–arterial) implanted within the first 24 hours after the transplant.

To estimate the left ventricle (LV) mass, we used the following equation, which has been published and validated in the literature [15].

$$\text{ Predicted LVmass }\,\left(g\right)=\alpha \times {\text{ height }}^{0.54}\times {\text{ weight }}^{0.61}$$

where α = 6.82 for women and 8.25 for men.

An independent correlation between each parameter and ECMO use in recipients was investigated. To determine whether there was a statistically significant difference between ECMO and non-ECMO groups for each parameter, we performed two-sample *t*-tests.

We used Harrell’s C statistic equivalent to the AU-ROC curve for dichotomous outcomes to validate the risk prediction equation. AU-ROC curve values of 0.7 to 0.8 were considered acceptable, as defined by Hosmer and Lemeshow [16].

The procedure respected TRIPOD guidelines for multivariate prediction models.

## 3. Results

### 3.1 Study Selection and Characteristics

**Fig. 1.**
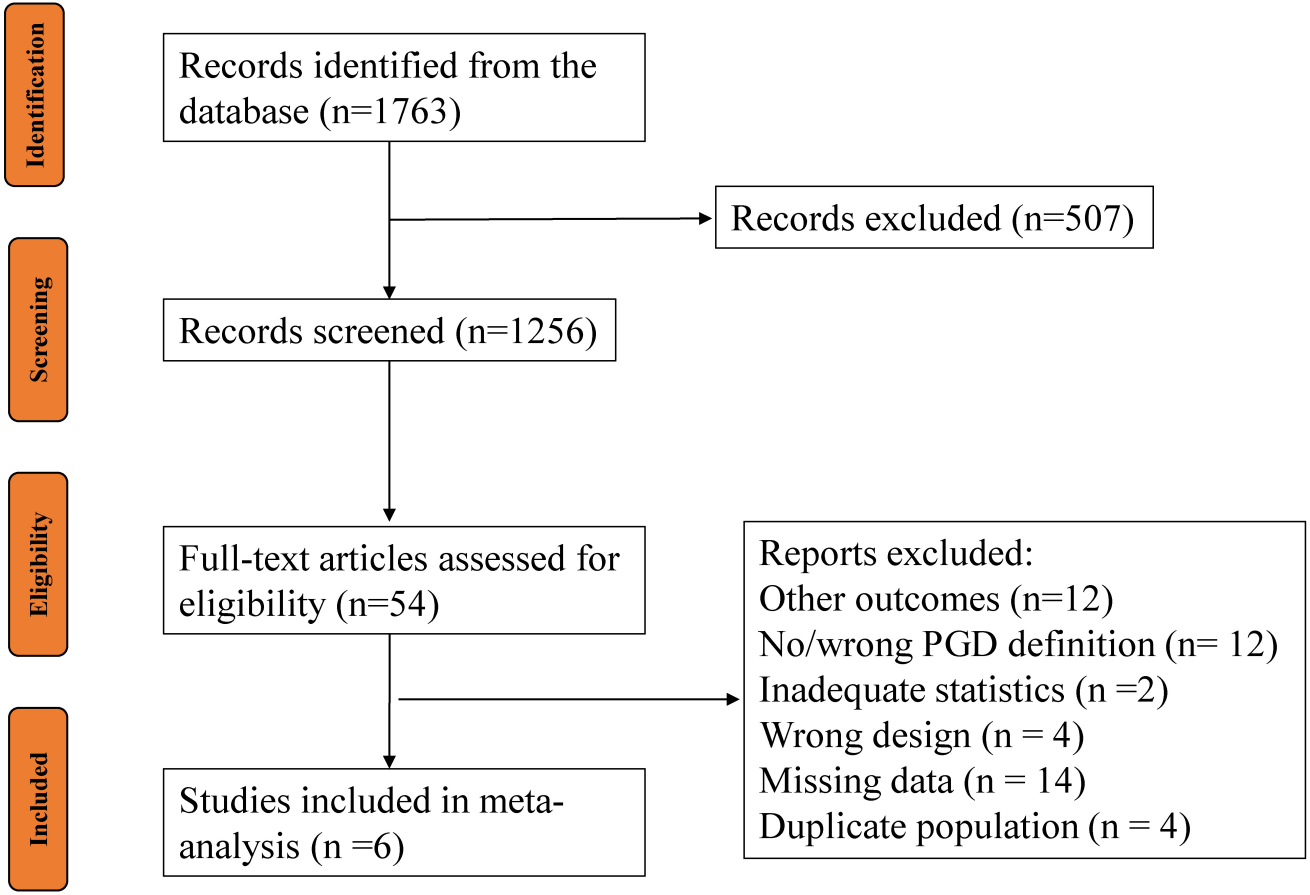
**Flowchart of study identification, inclusion, and exclusion**. PGD, primary graft dysfunction.

After screening 1256 publications, six studies were eligible for use to create the derivation cohort with adequate study sampling, statistical analysis, and reporting (Fig. 1).

Table 2 (Ref. [2, 8, 9, 17, 18, 19]) illustrates the demographics of studies considered for the derivation cohort. The cumulative number of patients considered was 10,528. The overall severe PGD incidence was 10.5% (95% CI: 5.3–12.4), and that of the severe PGD-related 30-day mortality was 38.6% (95% CI: 19.3–47.2). **Supplementary C** summarizes the quality assessment of individual studies.

**Table 2.** **Demographics of considered studies for the derivation cohort**.

Author Reference number	Year	Study type	Recruitment time frame	Cohort of patients	Donor data			Recipient data				Ischemic time minutes	Severe PGD	PGD-related mortality 95% CI
					% female	age	% sex	% female	age	% diabetes	% VAD			
							mismatch*							
Buchan TA	2021	Meta-analysis	1990–2020	8503	–	36 (23–50)	1870 (22%)	23	52 (30–69)	–	23%	186 (86–273)	654	255
*et al*. [9]													7.7%	39% (26%–53%)
Benck L	2021	Retrospective single center	2012–2018	734	242	34 (24–45)	84 (11.5%)	23	58 (50–66)	243 (33%)	18%	167	44	12
*et al*. [8]					32.9%								5.9%	28% (35%–65%)
King PM	2020	Retrospective single center	1992–2017	141	50	25 (20–32)	26 (18.4)	22	58 (51–63)	82 (14.4)	100%	143 (125–171)	32	6
*et al*. [19]					35.4%								22.6%	18.7%
Truby LK	2018	Retrospective single center	2009–2017	263	48	34.3 (21–60)	74 (28.1)	20	53.1	54 (20.5%)	100%	185 (106–201)	46	10
*et al*. [18]					18.2%								17.5%	21.7%
Nicoara A	2018	Retrospective single center	2009–2014	317	45	36 (26–46)	68 (21.4)	26	58 (48–63)	104 (32.8%)	33	180 (150–210)	20	–
*et al*. [2]					14.2%						9.8%		6.3%	
Rhee Y	2021	Retrospective single center	1992–2017	570	119	33.8 ± 11	50 (8.8)	28.9	46.7	82 (14.4%)	52	151.1 ± 59.5	29	1
*et al*. [17]					20.9%						9.1%		5.1%	3.4

*female donor to male recipient. PGD, primary graft dysfunction; VAD, ventricular assist device.

Table 3 summarizes the main characteristics of the derivation cohort.

**Table 3.** **Derivation cohort donor and recipient profiles**.

Variable	Donor	Recipient		*p*-value	
		Overall (n = 10,528)	Severe PGD (n = 732)	Donor *vs*. Recipient	Overall *vs*. PGD
Age (years)	38.6 ± 12.1	53 ± 16.2	60 ± 6.2	0.001	0.005
Female gender	3474 33%	3263 31%	202 28%	0.002	0.054
Gender mismatch	-	3127 29.7%	191 26.1%	-	0.038
Estimated LV mass mismatch (%)	-	4.13 ± 22.1	3.8 ± 22.9	-	0.031
Diabetes	2379 22.6%	3305 31.4%	287 39.2%	<0.001	0.001
Anemia	-	2210 21%	234 32%	-	<0.001
Decreased renal function	1326 12.6%	4000 38%	384 52.5%	<0.001	<0.001
Amiodarone	-	4453 42.3%	561 76.6%	-	<0.001
β-blocker	-	9212 87.5%	644 88%	-	0.705
VAD pre-HT	-	1558 14.8%	285 39%	-	0.001
Pulmonary hypertension (>40 mmHg)	-	2379 22.6%	189 25.8%	-	0.044
Mean total graft ischemic time (min)	-	182 ± 24	213 ± 62	-	<0.001
Mean bypass time (min)	-	125 ± 29	187 ± 79	-	0.004

PGD, primary graft dysfunction; LV, left ventricle; VAD, ventricular assist device; HT, heart transplant.

Variables with a *p*-value < 0.05 in the unadjusted analysis were included in the multivariable analysis. **Supplementary D** reports the subgroup outcome analyses.

### 3.2 Formula Predicting the Risk of PGD after HTx

**Fig. 2.**
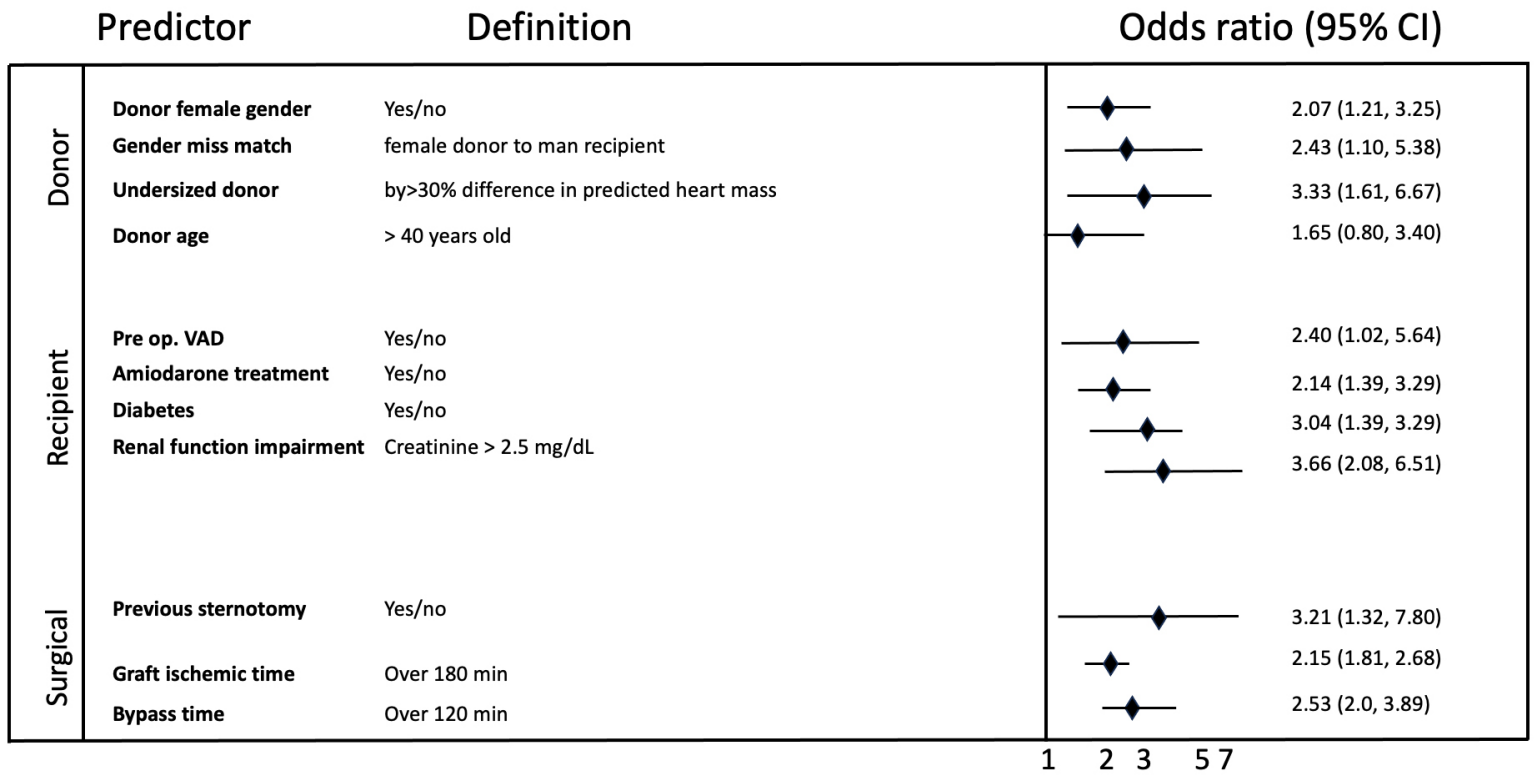
**Odds ratios of primary graft dysfunction predictors**. VAD, ventricular assist device.

Continuous data were stratified into categorical variables with reference categories. Eleven variables were identified in the multivariable analysis as significant PGD predictors. These variables and their odds ratios and regression coefficients are illustrated in Fig. 2.

We calculated the intercept and extracted the coefficients of each parameter from our logistic regression model (**Supplementary Material**), which provided the weight of each parameter in the model and allowed us to set the formula predicting severe PGD occurrence in the recipient. The intercept value was –2.98.

We defined a bench value for each continuous parameter to assign 0 or 1 in the formula if the parameter was below or above it, respectively (Table 4).

**Table 4.** **Continuous parameter values were used in the risk calculation formula**.

Variable	Values	
Value to enter in the formula	0/NO	1/YES
Donor’s age (years)	0 to 40	41 to 70
Renal function (creatinine mg/dL)	50 to 249	>250
Graft ischemic time (min)	<180	>181
Bypass time (min)	<120	>121

**Fig. 3.**
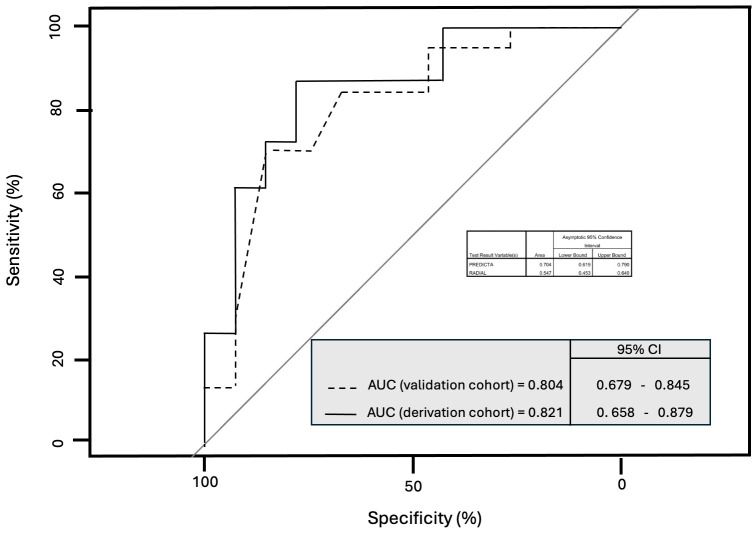
**Predictive performance of the GREF-11 formula evaluated using the C statistic, a term equivalent to the area under receiver operating characteristic (AU-ROC) curve**. The C statistic for the model constructed using the derivation cohort was 0.821 (continuous line), while the C statistic for the validation cohort was 0.804 (dotted line). GREF-11, graft dysfunction risk estimation formula; AUC, area under courbe.

After several attempts, the best predictive receiver operating characteristic (ROC) curve analysis (Fig. 3), corresponding to an accuracy of 0.821, included 11 parameters and was obtained via the formula as follows: for the binomial parameters, the coefficient was multiplied by 1 or 0 whether the medical condition was present in the donor/receiver or not. Regarding the sex of the donor, the coefficient was multiplied by 1 if the donor was male and by 0 if the donor was female.

### 3.3 Validation Cohort Characteristics. 

Between 2013 and 2022, 150 patients underwent HTxs at our institution, of which 116 met the inclusion criteria. The median age at HTx was 52.3 years, and the male gender was predominant 80 (69%). Major risk factors were smoking in 73 (62.9%), chronic obstructive pulmonary disease (COPD) in 11 (9.4%), and hypertension in 57 (49.1%). Heart failure etiology was ischemic disease in 54 (46.5%), hypertrophic in 8 (6.8%), dilated in 24 (20.6%), toxicity in 4 (3.4%), and other in 26 (22.4%) [20, 21]. The main donor, recipient, and procedural predictive variables are illustrated in Table 5. Overall, 29 recipients (25%) needed venous–arterial ECMO support within 24 hours post-HTx. Overall, 75% of the donors of recipients who needed ECMO were above 40 years old. The overall 30-day mortality was 4.3%, and 3.4% and 6.8% in the non-PGD and severe PGD groups, respectively.

**Table 5.** **Donor, recipient, and operative baseline characteristics in the validation cohort**.

	All	No-PGD	Severe PGD	*p*-value
	(n = 116)	(n = 87–75%)	(n = 29–25%)	
Gender mismatch	28 (24.1%)	12 (13.7%)	16 (55.1%)	<0.001
Donor age (years)	46 ± 14.4	39 ± 8	49 ± 8	<0.001
Donor female gender	52 (44.8%)	32 (36.7%)	20 (68.9%)	0.003
Undersized donor	29 (25%)	8 (9.2%)	21 (72.4%)	<0.001
VAD support	32 (27.5%)	28 (32.1%)	4 (13.7%)	0.055
Amiodarone treatment	51 (43.9%)	26 (29.8%)	25 (86.2%)	<0.001
Diabetes	26 (22.4%)	23 (26.4%)	3 (10.3%)	0.072
Renal function impairment	40 (34.4%)	22 (25.2%)	18 (62%)	<0.001
Pre-transplant anemia	44 (37.9%)	31 (35.6%)	13 (44.8%)	0.377
Pulmonary hypertension	39 (33.6%)	18 (20.6%)	21 (72.4%)	<0.001
Re-sternotomy	46 (39.6%)	29 (33.3%)	17 (58.6%)	0.016
Bypass time (min)	118 ± 29	102 ± 19	218 ± 32	<0.001
	75–216	75–191	130–275	
Graft total ischemic time (min)	123 ± 22	108 ± 29	203 ± 22	<0.001
	54–163	54–190	137–248	
Mortality at 30 days	5 (4.3%)	3 (3.4%)	2 (6.8%)	0.792

VAD, ventricular assistance device; PGD, primary graft dysfunction.

The model was applied to the validation cohort, and the ROC curve is reported in Fig. 2. The C statistic for the validation cohort was 0.804.

## 4. Discussion

Graft allocation has become a very complex process that HTx clinicians must experience. Despite the advances in scientific knowledge, there are still no international guidelines to help clinicians decide whether to accept or refuse the heart offered for a specific recipient. To address donor shortages, we have witnessed an expansion in the criteria for organ allocation, accepting transplants from older donors, diabetics, or those with transmissible diseases [22]. With the current demographic shift towards an increasingly older population and due to the advances in heart failure management, recipients are now more frail than before and receive complex medical treatments.

Sometimes, the situation is so complex that the decision-making process can be as challenging as the surgery. Indeed, clinicians have limited time to analyze a considerable amount of information and data, which is nevertheless necessary to estimate the impact their choice will likely have on the outcome of the recipient.

Inaccurate organ allocation could trigger PGD development. PGD has recently been classified according to a three-level grading system, mild, moderate, and severe, for a better comprehension of its pathophysiology and to provide a more systematic method for assessing treatment efficacy. The definition of mild and moderate categories relies on the requirement of inotropic support with a composite score, as described by Singh *et al*. [23]. The presence of severe myocardial dysfunction in the graft associated with severe hemodynamic impairment requiring extracorporeal short-term mechanical circulatory support in the form of ECMO, VADs, or causing the patient’s death allows diagnosis of severe PGD [3]. Notably, PGD usually occurs in the operating room after cardiopulmonary bypass (CPB) weaning fails.

In this study, we focused on severe PGD, as providing ECMO after HTx represents information that is clearly retrievable and not questionable in clinical studies, with severe PGD being an independent predictor of early death after HTx [23, 24]. In contrast, mild and moderate PGD were previously shown to have limited clinical impact on mortality [23, 24, 25].

For practical reasons, we favored a PGD definition on clinical grounds, regardless of the ventricle involved. We believe both left ventricle PGD and right ventricle PGD share similar risk factors, although the latter is not necessarily the consequence of severe pulmonary hypertension [26].

In the derivation cohort, we retrieved a pooled estimated incidence of severe PGD of 7.1%, with a related 30-day mortality of 38.6%.

Several authors have proposed a scoring system to identify patients most at risk of experiencing PGD [9, 10]. The risk score is a statistical tool based on multiple logistic regression models that use variables, or risk factors, to calculate an individual’s score and reflect the risk of a specific event. Existing scoring systems assign points to different risk factors based on their observed associations with the outcome. However, they may not capture complex interactions among variables as effectively as logistic regression. An essential issue in building a risk prediction model is the number of predictors that can be considered in the model-building process; a widely accepted recommendation suggests that at least 10 predictors should be considered per outcome event [17].

Three scoring systems have been validated to predict PGD after HTx: the RADIAL, PREDICTA, and ABCE, whereby the latter is the most recently validated [8]. The RADIAL score was developed by Segovia *et al*. [27] and consists of six risk factors derived from a multivariable analysis of 621 HTxs between 1984 and 2006, displaying a PGD incidence of 9%. The authors noted that an increased right atrial pressure exceeding 10 mmHg, recipient age over 60 years, recipient with diabetes mellitus, recipient with inotrope dependence, donor age over 30 years, and ischemic time length superior to 240 minutes were significant PGD risk factors. However, several authors did not find the RADIAL score predictive of PGD, particularly when considering severe PGD [2].

To improve the discriminatory value of the RADIAL score, Avtaar Singh *et al*. [28] proposed the PREDICTA score in 2019, in which bypass time and preoperative mechanical circulatory support replaced the central venous pressure and recipient with inotrope dependence parameters.

Recently, Benck *et al*. [8] proposed a risk score more focused on recipient and surgical parameters. In their multivariable analysis, these authors considered the following pharmacological treatments: Recipient treated with an angiotensin-converting enzyme inhibitor (ACEI), angiotensin receptor blocker (ARB), angiotensin receptor-neprilysin inhibitor (ARNI) plus mineralocorticoid receptor antagonist (MRA), amiodarone beta-blocker, as well as amiodarone (AMIO) treatment plus beta-blocker (BB). In addition, these authors identified four surgical factors, namely previous cardiac surgery, longer ischemic time, more red blood cell transfusions, and more platelet transfusions, which were shown to be associated with severe PGD [8].

Interestingly, the existing scoring systems, RADIAL, PREDICTA, and ABCE, only share one common parameter: graft total ischemic time. While diabetes and donor age appear in two of the three scores, most of the other risk factors are unique to each system. Each of these factors is supported by pathophysiological and statistical evidence, and while they hold predictive value, no single existing score integrates all these factors comprehensively. This fragmentation limits their applicability and can lead to inconsistent risk assessments. However, our approach was to consolidate the most predictive risk factors identified through a meta-analysis into a single robust logistic regression model. By doing so, we captured the complex interactions between variables, ensuring greater flexibility and interpretability. While scoring systems typically assign fixed points to individual risk factors, a risk calculator, such as the one we developed, uses a logistic regression model to dynamically assess the combined impact of multiple variables, providing a more nuanced and personalized risk prediction. This method allows us to maintain accuracy without the laborious calculations that typically make existing scoring systems impractical for routine clinical use. Therefore, our model was designed to provide a comprehensive and user-friendly tool that effectively predicts PGD risk, enhancing its clinical utility.

The model’s sensitivity (ability to predict true positives) and specificity (ability to predict true negatives) also depend on the number of variables considered. Therefore, the higher the number of variables included in the model, the higher the model’s specificity and accuracy. Our selected approach was to systematically analyze each organ allocation process step to identify as many predictors of severe PGD as possible and their weight in predicting the event to create a formula for risk prediction with the highest discriminatory value. Our formula considered 11 variables obtained by analyzing the largest presently available patient cohort and only considered routinely available or easily quantified parameters to avoid missing data and potential inaccuracies in scoring system predictions.

Incorporating the GREF-11 into clinical practice would offer a dual approach to improving the outcomes of high-risk patients. Firstly, it would allow clinicians to identify cases where the predicted PGD risk is particularly high. In such scenarios, it may be advisable to reconsider the transplant or to seek alternative donor organs to avoid proceeding with a transplant under unfavorable conditions. Secondly, for patients identified as high-risk who proceed with the transplant, it would enable proactive planning for postoperative management. More specifically, early implantation of ECMO can be considered to mitigate PGD impact and support the graft during the critical early postoperative period.

The drawback of our approach is that much data must be considered and that risk computation results in a complex and time-consuming calculation, potentially discouraging its clinical use. Therefore, we developed a user-friendly smartphone application to overcome this potential limitation and render the risk calculation adapted to physicians.

### 4.1 Donor Factors

#### 4.1.1 Undersized Donor 

Undersized donors, defined as >30% difference in donor and recipient predicted heart mass, were associated with a 3.3-fold increase in odds ratio. Several authors agreed with the observation that this is an independent and strong predictor of severe PGD. This finding is consistent with the hypothesis that a heart that is too small cannot satisfy the recipient’s hemodynamic requirements [9, 29]. This parameter was likely more accurate with respect to size and weight mismatch, as it serves as an indicator of anatomical and functional compatibility.

However, some studies have questioned the role of this risk factor in predicting severe PGD [10, 28], probably because the correlation between mismatch in predicted heart mass index and PGD is logarithmic rather than linear. This means that this parameter has no predicting value when a difference is within 30%, whereas above 30%, the parameter becomes a strong predictor of severe PGD. It is worth noting that we have considered this aspect when preparing the risk calculator.

#### 4.1.2 Donor Gender and Gender Mismatch

Female donor and female donor to male receiver gender mismatch are risk factors for PGD, both with a 2.4-fold increase in odds ratio. However, the exact mechanism is poorly understood, as it persisted despite appropriate organ matching.

#### 4.1.3 Donor Age

Older age (>40 or >50 years old, depending on the study) was shown to be associated with a decrease in recipient survival [24, 30]. This may be due to poorer tolerance for longer ischemic times in hearts from older donors, as previously reported [24, 26, 30]. However, the weight of this variable differs in the existing score risks, given that some authors stratified donor age into four age classes of 10 years, assuming that each decade increment in donor age increases PGD odds by 20% [31]. Others defined a limited age to associate odds [27]. Our statistical analysis defined 40 years of age as the cut-off value, as donors older than 40 displayed a 1.6-fold PGD risk. Once again, we could speculate that the correlation between different ages and PGD is logarithmic rather than linear, meaning that this parameter has no predicting value when the age is below 40 years.

### 4.2 Recipient Factors

#### 4.2.1 Preoperative Mechanical Circulatory Support

Several studies identified the preoperative ECMO/VAD support in HTx recipients as a risk factor for PGD [24, 31, 32, 33, 34]. Truby *et al*. [18] reported a significant association in patients on Continuous Flow Left Ventricular Assist Device (CF-LVAD) support for more than a year. Short-term ECMO and long-term VAD circulatory support have been identified as independent risk factors for PGD, both resulting in a 2.4-fold increase in risk. The pathogenesis remains unclear, given that patients requiring mechanical circulatory support usually exhibit other factors that could play a role in PGD occurrence, such as exposure to amiodarone, displaying high creatinine levels, and spending longer on waiting lists [35].

Our multivariate analysis suggested that LVAD support was independently associated with PGD following HTx, with a 2.4-fold increase in odds.

#### 4.2.2 Diabetes Mellitus 

Diabetes mellitus in recipients plays a role in PGD, as extensively outlined by several clinical studies [2, 27]. However, the exact mechanism is not well understood. Possible reasons for such associations include changes in endothelial permeability, excessive vascular protein deposition, altered blood flow in diabetic recipients secondary to direct glucose-mediated endothelial damage, oxidative stress from superoxide overproduction, and production of advanced glycation end-products [36]. In our meta-analysis, recipient diabetes was associated with a 3-fold increased risk of severe PGD [5].

#### 4.2.3 Re-Sternotomy

Recipient re-sternotomy was associated with an almost 3-fold increased PGD risk in one cohort [37], which was confirmed by our analysis. One possible explanation for this observation is the increased technical difficulty of such procedures; hence, the associated longer cardiopulmonary bypass and ischemic times, higher bleeding rates, re-exploration, and blood transfusion.

#### 4.2.4 Preoperative Amiodarone Therapy

Some authors have indicated a dose- or duration-dependent association between amiodarone use and PGD, even though the underlying pathophysiological mechanism remains unknown [38]. One hypothesis suggests that amiodarone can enter the graft, potentially exerting negative chronotropic and inotropic effects via calcium channel inhibition and β-receptor blockade [39]. In our meta-analysis, we have not stratified the results according to treatment dose and length, whereas amiodarone exerted a low impact on PGD, increasing its risk by only 1.04 times. Nevertheless, we included the drug in our risk prediction equation to improve accuracy.

#### 4.2.5 High Creatinine Levels

Preoperative renal function impairment has been associated with PGD [9], and our results reasonably confirmed this association. Buchan *et al*. [9] found that a 1 mg/dL increase in recipient creatinine levels was associated with a 3.6-fold increase in odds ratio. However, the authors were unable to explain the pathophysiological background of their findings. We could not perform a similar detailed analysis using data from the derivation cohort. More specifically, we found that a creatinine level above twice the normal values was associated with a 3.66-fold increase in PGD risk.

### 4.3 Procedural Factors

#### 4.3.1 Total Ischemic Time

Several studies reported that prolonged ischemic time increased PGD risk and 30-day mortality post-HTx [15, 24, 25]. In contrast, the 4-hour cut-off value on which there is a clear consensus, as reported in the RADIAL score, is of poor help in stratifying the PGD risk because almost all organ allocations respect this criterion. In particular, warm ischemic time exceeding 80 minutes is considered an independent risk factor for 30-day mortality [40, 41].

However, ischemic time is also closely related to donor characteristics, as older donors with LV hypertrophy and hypertension are more susceptible to ischemic injury [31, 42]. In our analysis, we could not stratify the risk according to warm or cold ischemic time and donor age due to missing data. In contrast, we attained good predicting value when the total ischemic time exceeded 180 minutes, with a 2.15-fold increase in severe PGD risk. Therefore, when entering data in the formula, one should check if the expected total ischemic time will significantly exceed 180 minutes.

#### 4.3.2 CPB Time

Several studies have reported potential associations between cardiopulmonary bypass times and PGD development [28, 43]. While the exact mechanism remains unclear, according to one hypothesis, this association relies on the initiation of a systemic inflammatory response through the contact of blood with foreign surfaces in the CPB circuit, leading to inflammatory pathway activation, cytokine release, generation of oxygen-free radicals, and eventually vasoplegia with decreased systemic vascular resistances [43]. CPB time prolongations are also associated with increased blood transfusion use, secondary to coagulopathy and hemolysis, which are associated with infection, ischemic postoperative morbidity, as well as increased early and late mortality [44].

We found a 2.53-fold increase in severe PGD when the CPB time exceeded 120 minutes. Therefore, when entering data in the formula, one should check if the expected CPB time will significantly exceed 120 minutes.

Three more variables are considered to impact severe PGD, yet no predictive value is displayed in our analysis: blood transfusion, recipient age, and pulmonary hypertension. Moreover, we could not consider biological parameters such as those included in the ABCE score due to a lack of data in our derivation cohort.

The identified parameters were integrated into an 11-variable formula called the graft risk estimation formula or GREF-11. To our knowledge, this is the most comprehensive formula for predicting severe PGD after HTx. The weight of each variable introduced into the GREF-11 was determined using multivariate analysis of the validation cohort data. Moreover, one unique feature of our model is that the intercept (β0) of the equation had a negative value of –2.98—the intercept represents where the logistic curve crosses the ordinate axis when all independent variables equal zero. In the context of predicting a negative event, such as PGD, a negative intercept value might suggest a prediction of a negative outcome even in the absence of all risk factors. This is why our model predicts a 4.8% risk of severe PGD, even when all parameters point to a smooth outcome.

The ability of the model to identify patients most at risk of developing severe PGD is expressed by the AU-ROC curve. Indeed, the AU-ROC value of 0.89 indicates that our score predicts severe PGD more accurately than any previously reported scoring system. In a comparative study by Singh *et al*. [23], the PREDICTA score could accurately predict PGD with a better predictive function than the RADIAL score and a better discriminatory value (AU-ROC: 0.740 *vs*. 0.547).

### 4.4 Cohort Comparisons 

We used our past clinical experience as the validation cohort for the model. To ensure data applicability, the demographic data of both cohorts were compared, and no statistically significant differences regarding demographic and comorbidity variables were observed between the patient derivation and validation cohorts. However, severe PGD and mortality incidence were significantly different, given that the PGD incidence in the derivation cohort was 10.5% versus 25% in the validation cohort, with 30-day mortality of 38.6% and 6.8%, respectively. This apparent contradiction can be accounted for by the more aggressive strategy we adopted to treat graft dysfunction in the operative phase compared with that commonly reported in the literature. Due to organ shortages, we tended to accept grafts from likely marginal donors, thereby increasing the risk of including dysfunctional hearts. We were more prone to use short-term mechanical circulatory support in patients with the pharmacological backing rapidly and progressively increased to very high doses. We believe that high doses of inotropic drugs and vasoconstrictor agents challenge the cardiocirculatory system and that these drugs could exhibit deleterious effects on graft dysfunction recovery, whereas ECMO provides better conditions for graft recovery.

While some parameters (e.g., donor female gender, gender mismatch, amiodarone treatment, undersized donor, donor age, and renal function impairment) were statistically significant in the derivation cohort but not in the validation cohort, this is a common outcome in model validation. Differences in patient characteristics and statistical power between the cohorts can lead to variability in significance. Importantly, these parameters remain clinically relevant and were included in the final model based on their demonstrated value in the derivation cohort and alignment with clinical knowledge. The primary role of the validation cohort is to confirm the overall robustness and predictive accuracy of the model rather than ensure every parameter retains significance across both cohorts.

The 30-day mortality rate in the validation cohort was 5-fold lower than in the derivation cohort, thereby supporting the hypothesis that aggressively used ECMO was likely effective in facilitating graft recovery in severe PGD, thereby minimizing its effects on early mortality. Although this was not the aim of this study, we can speculate that early ECMO use could improve the clinical outcome of patients with PGD post-HTx in a superior manner to isolated pharmacological treatments.

Although the validation cohort was small, limiting statistical power to detect significant differences in mortality, including a broad range of parameters, enhances the overall predictive accuracy of the model by accounting for the complex factors contributing to PGD. We acknowledge that this may seem complex, but the score has been designed to remain clinically practical and applicable without causing confusion.

**Fig. 4.**
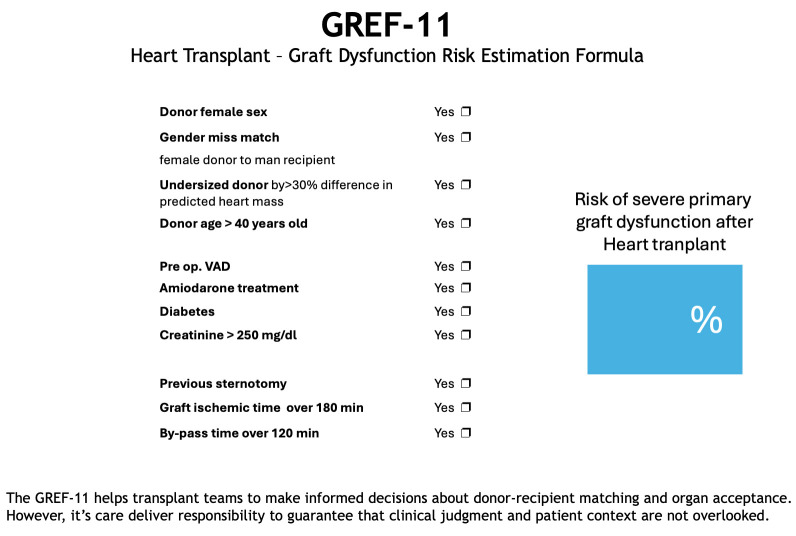
**Layout of the mobile application**. GREF-11, graft dysfunction risk estimation formula; VAD, ventricular assistance device.

As mentioned before, managing huge amounts of data is time-consuming and laborious unless there is consistent help from technologies such as mobile applications. Mobile applications are being increasingly utilized in healthcare, many of which focus on patient education, health behaviors, and disease management. This free application can be used on smartphones and tablets (Fig. 4), is available for Apple and Android devices, and can be downloaded from https://gref-11.com or using the following QR code.

### 4.5 Study Limitations

This study has limitations. Most of the studies included in the meta-analysis were retrospective studies, as were the data from our experience, thus introducing a methodology bias because model performance largely depends on the accuracy of data and variables collected.

It is important to acknowledge that the studies included in our meta-analysis span several years, during which significant advancements in technology, particularly in VADs and organ preservation, occurred. These technological changes may have influenced the outcomes observed during different periods. Although our analysis did not stratify results based on these technological eras, this represents a limitation in our study. Future research should consider stratifying data to evaluate the impact of these technological advancements on the identified risk factors.

The formula has not been developed for donors after circulatory arrest. With the increasing use of DCD worldwide, we should implement accurate, standardized definitions regarding the start and end of meaningful warm ischemia time to enable important comparisons among studies with respect to PGD risks. Moreover, the proposed model is purely explanatory, requiring external validation and cross-validation in a prospective cohort.

While our study focused primarily on developing and validating the predictive accuracy of the model, we did not include a decision curve analysis (DCA), which is a valuable tool for assessing the clinical utility of predictive models by evaluating net benefits across various decision thresholds. To understand the importance of the DCA in considering how this model could inform clinical decision-making, we thus suggest that future research incorporate this analysis.

## 5. Conclusions

The surgical decision-making process in personalized medicine should evolve from individual clinician judgments and center experiences to a reproductive and reliable personalized surgical risk prediction method. The GREF-11 tool presented in this study should offer benefits, including standardized risk assessment and clinical decision support, and it is readily available to clinicians at the bedside. However, this score must undergo further validation. Furthermore, future studies should focus on validating the GREF-11 score across diverse patient populations and various clinical settings to ensure its reliability and applicability in contemporary clinical practice.

## Availability of Data and Materials

The datasets used and/or analyzed during the current study are available from the corresponding author on reasonable request.

## Supplementary Material

Supplementary material associated with this article can be found, in the online version, at https://doi.org/10.31083/RCM25170.

## References

[1] Khush KK, Hsich E, Potena L, Cherikh WS, Chambers DC, Harhay MO (2021). The International Thoracic Organ Transplant Registry of the International Society for Heart and Lung Transplantation: Thirty-eighth adult heart transplantation report - 2021; Focus on recipient characteristics. *The Journal of Heart and Lung Transplantation: the Official Publication of the International Society for Heart Transplantation*.

[2] Nicoara A, Ruffin D, Cooter M, Patel CB, Thompson A, Schroder JN (2018). Primary graft dysfunction after heart transplantation: Incidence, trends, and associated risk factors. *American Journal of Transplantation: Official Journal of the American Society of Transplantation and the American Society of Transplant Surgeons*.

[3] Kobashigawa J, Zuckermann A, Macdonald P, Leprince P, Esmailian F, Luu M (2014). Report from a consensus conference on primary graft dysfunction after cardiac transplantation. *The Journal of Heart and Lung Transplantation: the Official Publication of the International Society for Heart Transplantation*.

[4] Lima EB, Cunha CRD, Barzilai VS, Ulhoa MB, Barros MRD, Moraes CS (2015). Experience of ECMO in primary graft dysfunction after orthotopic heart transplantation. *Arquivos Brasileiros De Cardiologia*.

[5] Sabatino M, Vitale G, Manfredini V, Masetti M, Borgese L, Maria Raffa G (2017). Clinical relevance of the International Society for Heart and Lung Transplantation consensus classification of primary graft dysfunction after heart transplantation: Epidemiology, risk factors, and outcomes. *The Journal of Heart and Lung Transplantation: the Official Publication of the International Society for Heart Transplantation*.

[6] Nicoara A, Kretzer A, Cooter M, Bartz R, Lyvers J, Patel CB (2020). Association between primary graft dysfunction and acute kidney injury after orthotopic heart transplantation - a retrospective, observational cohort study. *Transplant International: Official Journal of the European Society for Organ Transplantation*.

[7] Squiers JJ, DiMaio JM, Van Zyl J, Lima B, Gonzalez-Stawisnksi G, Rafael AE (2021). Long-term outcomes of patients with primary graft dysfunction after cardiac transplantation. *European Journal of Cardio-thoracic Surgery: Official Journal of the European Association for Cardio-thoracic Surgery*.

[8] Benck L, Kransdorf EP, Emerson DA, Rushakoff J, Kittleson MM, Klapper EB (2021). Recipient and surgical factors trigger severe primary graft dysfunction after heart transplant. *The Journal of Heart and Lung Transplantation: the Official Publication of the International Society for Heart Transplantation*.

[9] Buchan TA, Moayedi Y, Truby LK, Guyatt G, Posada JD, Ross HJ (2021). Incidence and impact of primary graft dysfunction in adult heart transplant recipients: A systematic review and meta-analysis. *The Journal of Heart and Lung Transplantation: the Official Publication of the International Society for Heart Transplantation*.

[10] Villa BP, Alotaibi S, Brozzi N, Spindler KP, Navia J, Hernandez-Montfort J (2022). Prognostic value of patient-reported outcome measures in adult heart-transplant patients: a systematic review. *Journal of Patient-reported Outcomes*.

[11] Foroutan F, Iorio A, Thabane L, Guyatt G (2020). Calculation of absolute risk for important outcomes in patients with and without a prognostic factor of interest. *Journal of Clinical Epidemiology*.

[12] Page MJ, McKenzie JE, Bossuyt PM, Boutron I, Hoffmann TC, Mulrow CD (2021). The PRISMA 2020 statement: an updated guideline for reporting systematic reviews. *BMJ (Clinical Research Ed.)*.

[13] Tripepi G, Heinze G, Jager KJ, Stel VS, Dekker FW, Zoccali C (2013). Risk prediction models. *Nephrology, Dialysis, Transplantation: Official Publication of the European Dialysis and Transplant Association - European Renal Association*.

[14] Hosmer DW, Lemeshow S, Sturdivant RX (2013). *Applying logistic regression*.

[15] Bluemke DA, Kronmal RA, Lima JA, Liu K, Olson J, Burke GL (2008). The relationship of left ventricular mass and geometry to incident cardiovascular events: the MESA (Multi-Ethnic Study of Atherosclerosis) study. *Journal of the American College of Cardiology*.

[16] Cook NR (2007). Use and misuse of the receiver operating characteristic curve in risk prediction. *Circulation*.

[17] Rhee Y, Kim HJ, Kim JJ, Kim MS, Lee SE, Yun TJ (2021). Primary Graft Dysfunction After Isolated Heart Transplantation - Incidence, Risk Factors, and Clinical Implications Based on a Single-Center Experience. *Circulation Journal: Official Journal of the Japanese Circulation Society*.

[18] Truby LK, Takeda K, Topkara VK, Takayama H, Garan AR, Yuzefpolskaya M (2018). Risk of severe primary graft dysfunction in patients bridged to heart transplantation with continuous-flow left ventricular assist devices. *The Journal of Heart and Lung Transplantation: the Official Publication of the International Society for Heart Transplantation*.

[19] King PM, Raymer DS, Shuster J, Crain M, Bhatia A, Hartupee J (2020). Right Heart Failure While on Left Ventricular Assist Device Support Is Associated with Primary Graft Dysfunction. *ASAIO Journal (American Society for Artificial Internal Organs: 1992)*.

[20] Hullin R, Abdurashidova T, Pitta-Gros B, Schukraft S, Rancati V, Lu H (2023). Post-transplant survival with pre-transplant durable continuous-flow mechanical circulatory support in a Swiss cohort of heart transplant recipients. *Swiss Medical Weekly*.

[21] Anouck Z, Tozzi P, Regamey J, Abdurashidova T, Meyer P, Lefol K (2022). Has the profile of heart transplantation recipients changed within the last three decades?. *Swiss Medical Weekly*.

[22] Hsich EM (2016). Matching the Market for Heart Transplantation. *Circulation. Heart Failure*.

[23] Singh SSA, Dalzell JR, Berry C, Al-Attar N (2019). Primary graft dysfunction after heart transplantation: a thorn amongst the roses. *Heart Failure Reviews*.

[24] D’Alessandro C, Golmard JL, Barreda E, Laali M, Makris R, Luyt CE (2011). Predictive risk factors for primary graft failure requiring temporary extra-corporeal membrane oxygenation support after cardiac transplantation in adults. *European Journal of Cardio-thoracic Surgery: Official Journal of the European Association for Cardio-thoracic Surgery*.

[25] Squiers JJ, Saracino G, Chamogeorgakis T, MacHannaford JC, Rafael AE, Gonzalez-Stawinski GV (2017). Application of the International Society for Heart and Lung Transplantation (ISHLT) criteria for primary graft dysfunction after cardiac transplantation: outcomes from a high-volume centre†. *European Journal of Cardio-thoracic Surgery: Official Journal of the European Association for Cardio-thoracic Surgery*.

[26] Lim HS, Ranasinghe A, Quinn D, Chue CD, Mascaro J (2021). Pathophysiology of severe primary graft dysfunction in orthotopic heart transplantation. *Clinical Transplantation*.

[27] Segovia J, Cosío MDG, Barceló JM, Bueno MG, Pavía PG, Burgos R (2011). RADIAL: a novel primary graft failure risk score in heart transplantation. *The Journal of Heart and Lung Transplantation: the Official Publication of the International Society for Heart Transplantation*.

[28] Avtaar Singh SS, DAS DE S, Rushton S, Berry C, Al-Attar N (2019). PREDICTA: A Model to Predict Primary Graft Dysfunction After Adult Heart Transplantation in the United Kingdom. *Journal of Cardiac Failure*.

[29] Reed RM, Netzer G, Hunsicker L, Mitchell BD, Rajagopal K, Scharf S (2014). Cardiac size and sex-matching in heart transplantation: size matters in matters of sex and the heart. *JACC. Heart Failure*.

[30] Khush KK, Potena L, Cherikh WS, Chambers DC, Harhay MO, Hayes D (2020). The International Thoracic Organ Transplant Registry of the International Society for Heart and Lung Transplantation: 37th adult heart transplantation report-2020; focus on deceased donor characteristics. *The Journal of Heart and Lung Transplantation: the Official Publication of the International Society for Heart Transplantation*.

[31] Russo MJ, Chen JM, Sorabella RA, Martens TP, Garrido M, Davies RR (2007). The effect of ischemic time on survival after heart transplantation varies by donor age: an analysis of the United Network for Organ Sharing database. *The Journal of Thoracic and Cardiovascular Surgery*.

[32] Russo MJ, Iribarne A, Hong KN, Ramlawi B, Chen JM, Takayama H (2010). Factors associated with primary graft failure after heart transplantation. *Transplantation*.

[33] Hong KN, Iribarne A, Worku B, Takayama H, Gelijns AC, Naka Y (2011). Who is the high-risk recipient? Predicting mortality after heart transplant using pretransplant donor and recipient risk factors. *The Annals of Thoracic Surgery*.

[34] Awad M, Czer LSC, Mirocha J, Ruzza A, Rafiei M, Reich H (2015). Prior sternotomy increases the mortality and morbidity of adult heart transplantation. *Transplantation Proceedings*.

[35] See SB, Clerkin KJ, Kennel PJ, Zhang F, Weber MP, Rogers KJ (2017). Ventricular assist device elicits serum natural IgG that correlates with the development of primary graft dysfunction following heart transplantation. *The Journal of Heart and Lung Transplantation: the Official Publication of the International Society for Heart Transplantation*.

[36] Taghavi S, Jayarajan SN, Wilson LM, Komaroff E, Testani JM, Mangi AA (2013). Cardiac transplantation can be safely performed using selected diabetic donors. *The Journal of Thoracic and Cardiovascular Surgery*.

[37] Still S, Shaikh AF, Qin H, Felius J, Jamil AK, Saracino G (2018). Reoperative sternotomy is associated with primary graft dysfunction following heart transplantation. *Interactive Cardiovascular and Thoracic Surgery*.

[38] Wright M, Takeda K, Mauro C, Jennings D, Kurlansky P, Han J (2017). Dose-dependent association between amiodarone and severe primary graft dysfunction in orthotopic heart transplantation. *The Journal of Heart and Lung Transplantation: the Official Publication of the International Society for Heart Transplantation*.

[39] Jennings DL, Martinez B, Montalvo S, Lanfear DE (2015). Impact of pre-implant amiodarone exposure on outcomes in cardiac transplant recipients. *Heart Failure Reviews*.

[40] Marasco SF, Kras A, Schulberg E, Vale M, Lee GA (2012). Impact of warm ischemia time on survival after heart transplantation. *Transplantation Proceedings*.

[41] Banner NR, Thomas HL, Curnow E, Hussey JC, Rogers CA, Bonser RS (2008). The importance of cold and warm cardiac ischemia for survival after heart transplantation. *Transplantation*.

[42] Wong G, Teixeira-Pinto A, Chapman JR, Craig JC, Pleass H, McDonald S (2017). The Impact of Total Ischemic Time, Donor Age and the Pathway of Donor Death on Graft Outcomes After Deceased Donor Kidney Transplantation. *Transplantation*.

[43] Prieto D, Correia PM, Batista M, Antunes MJ (2018). Primary graft failure after cardiac transplantation: prevalence, prognosis and risk factors. *Interactive Cardiovascular and Thoracic Surgery*.

[44] Murphy GJ, Reeves BC, Rogers CA, Rizvi SIA, Culliford L, Angelini GD (2007). Increased mortality, postoperative morbidity, and cost after red blood cell transfusion in patients having cardiac surgery. *Circulation*.

