# Supplementary material for: Risk Estimation of Severe Primary Graft Dysfunction in Heart Transplant Recipients Using a Smartphone
Source: Rev Cardiovasc Med. 2025 Jan 8;26(1):25170. doi: 10.31083/RCM25170 (PMC11759961; doi:10.31083/RCM25170)
Supplement: Supplementary file 1 [file 2153-8174-26-1-25170-s1.zip › Informed consent.pdf]

Déclaration de consentement pour l'utilisation des données de santé et des échantillons à des fins de recherche

Nom et prénom

Date de naissance

Ⓐ J'accepte que mes données de santé et mes échantillons biologiques résiduels collectés durant les soins (consultations ambulatoires et hospitalisations) soient conservés, transmis et utilisés à des fins de recherche.

❑ OUI ❑ NON

Si vous avez coché « OUI », veuillez répondre à la proposition B. Si vous avez coché « NON », passez directement au point C.

Ⓑ J'accepte de donner un échantillon de sang supplémentaire de 7,5 mL pour la Biobanque génomique du CHUV à des fins d'analyses génétiques pour la recherche.

- Un prélèvement supplémentaire de 7,5 mL de sang pourra être effectué à l'occasion de mes soins au CHUV.
- Ce prélèvement permettra de faire des analyses génétiques à des fins de recherche.

❑ OUI ❑ NON

Quelle que soit votre réponse, veuillez passer au point C.

Ⓒ Confirmation de ma décision

J'ai compris :

- les explications sur la réutilisation de mes données cliniques et échantillons biologiques à des fins de recherche, détaillées dans la brochure d'information;
- que je suis libre de contacter l'Unité du consentement à la recherche du CHUV aux coordonnées indiquées au bas de ce formulaire, ou un.e professionnel.le de santé en charge de mes soins au CHUV pour obtenir de plus amples informations et explications;
- que mes données personnelles sont protégées et qu'elles ne seront utilisées pour la recherche que de manière codée ou anonymisée;
- que mes données et échantillons biologiques peuvent être utilisés dans des projets de recherche nationaux et internationaux, dans les secteurs public et privé;
- que les projets peuvent inclure des analyses génétiques sur mes échantillons, à des fins de recherche;
- que je pourrais être recontacté.e dans le cas où des résultats pertinents pour ma santé seraient mis en évidence;
- que ma décision est volontaire et n'a pas d'effet sur mon traitement médical;
- que ma décision est valable pour une durée illimitée à moins que je retire mon consentement;
- que je peux retirer mon consentement à n'importe quel moment sans avoir à justifier ma décision;
- que si je coche « NON » au point A en signant cette déclaration, mes données cliniques et échantillons biologiques ne pourront pas être utilisés pour la recherche;
- que si je ne signe pas la déclaration de consentement (absence de réponse), la loi prévoit que mes données et échantillons pourront exceptionnellement être utilisés si la commission d'éthique compétente donne son autorisation spéciale.

Lieu et date

Signature de la patiente ou du patient

En cas de question ou de remarque, n'hésitez pas à nous contacter.

Département de la formation et recherche
Unité du consentement à la recherche
Boîte aux lettres N°47
Rue du Bugnon 21, 1011 Lausanne
021 314 18 78 - info.cg@chuv.ch

CHUV V2.3/0.1 du 15 mars 2021
AMT0810 - Consentement général
