# Supplementary material for: Risk Estimation of Severe Primary Graft Dysfunction in Heart Transplant Recipients Using a Smartphone
Source: Rev Cardiovasc Med. 2025 Jan 8;26(1):25170. doi: 10.31083/RCM25170 (PMC11759961; doi:10.31083/RCM25170)
Supplement: Supplementary file 1 [file 2153-8174-26-1-25170-s1.zip › Supplementary A Study Eligibility Form.pdf]

Appendix A   Study Eligibility Form

**Article ID**	
Author: Year Journal Title	
**Population**	
Heart Transplant Recipients	YES ☐ NO ☐
Adults (> 18 years old)	YES ☐ NO ☐
**Predictor[1]**	
Any predictor of PGD	YES ☐ NO ☐
**Adjusted Analysis[2]**	
Multivariate analysis	YES ☐ NO ☐
**Outcomes reported**	
Severe PGD	YES ☐ NO ☐
30-day mortality after heart transplant	YES ☐ NO ☐
**Type of article[2]**	
Cohort study (retrospective or prospective) or	YES ☐ NO ☐
RCT cohort	YES ☐ NO ☐
Meta-analysis on predictors	YES ☐ NO ☐
**Duplicated population**	
Does this study provide new information?	YES ☐ NO ☐
Is study more recent?	YES ☐ NO ☐
**Study inclusion**	
All the answers are YES	INCLUDE
Any answer is NO	EXCLUDE

Instructions

[1]Consider YES if any type of predictor including clinical characteristics, laboratory values, test results and any other clinical event. Exclude study evaluating therapies as predictor and not reporting on any other potential predictor.

[2]Consider NO if the study used only any other type of adjustment for potential confounders, including matched design or stratification.

[3]Meta-analysis on observational studies evaluating predictors could be included in the individual studies used multivariable analysis
