# Supplementary material for: Risk Estimation of Severe Primary Graft Dysfunction in Heart Transplant Recipients Using a Smartphone
Source: Rev Cardiovasc Med. 2025 Jan 8;26(1):25170. doi: 10.31083/RCM25170 (PMC11759961; doi:10.31083/RCM25170)
Supplement: Supplementary file 1 [file 2153-8174-26-1-25170-s1.zip › Supplementary B Data Abstraction Form.pdf]

Appendix B – Data Abstraction Form

(A) STUDY IDENTIFICATION

Reviewer PT ☐ SAT ☐ EH ☐
Article title
First Author
Journal
Publication year
Single center ☐ Multicenter ☐ Countries involved

(B) STUDY CHARACTERISTICS

Study design Prospective ☐ Retrospective ☐ Randomized ☐ Meta-analysis ☐
Outcomes 30-day mortality ☐ PGD ☐ severe PGD ☐ ECMO ☐

(C) POPULATION

Number of patient included _______ Not reported ☐
Inclusion criteria Not reported ☐
Number of events Deaths ______ PGD ______ Severe PGD _______

Recipient baseline characteristics

	Mean ☐ Median ☐ SD ☐ SE ☐ IQR ☐ Range ☐ Not reported ☐
Age	
Gender	
Gender mismatch	
Estimated	
LV mass mismatch	
BMI	
Diabetes	
Anemia	
Renal impairment	
Amiodarone	
B-blocker	
PHT	
VAD pre-transplant	
Graft ischemic time	
Bypass time	

Donor baseline characteristics

	Mean ☐ Median ☐ SD ☐ SE ☐ IQR ☐ Range ☐ Not reported ☐
Age	
Donor female	
Undersized donor	

(D) PREDICTORS

Outcome: severe PGD and related 30-day mortality

Categorical variables

Name:

Define comparative groups Numerator:	Denominator:	
☐ HR ☐ OR ☐ RR		☐ not reported
☐ 95% CI		☐ not reported
If HR/OR not reported, was the association statistically significant?	☐Yes ☐ No	☐ not reported

Name:

Define comparative groups Numerator:	Denominator:	
☐ HR ☐ OR ☐ RR		☐ not reported
☐ 95% CI		☐ not reported
If HR/OR not reported, was the association statistically significant?	☐Yes ☐ No	☐ not reported

Name:

Define comparative groups Numerator:	Denominator:	
☐ HR ☐ OR ☐ RR		☐ not reported
☐ 95% CI		☐ not reported
If HR/OR not reported, was the association statistically significant?	☐Yes ☐ No	☐ not reported

Name:

Define comparative groups Numerator:	Denominator:	
☐ HR ☐ OR ☐ RR		☐ not reported
☐ 95% CI		☐ not reported
If HR/OR not reported, was the association statistically significant?	☐Yes ☐ No	☐ not reported

Name:

Define comparative groups Numerator:	Denominator:	
☐ HR ☐ OR ☐ RR		☐ not reported
☐ 95% CI		☐ not reported
If HR/OR not reported, was the association statistically significant?	☐Yes ☐ No	☐ not reported

Name:

Define comparative groups Numerator:	Denominator:	
☐ HR ☐ OR ☐ RR		☐ not reported
☐ 95% CI		☐ not reported
If HR/OR not reported, was the association statistically significant?	☐Yes ☐ No	☐ not reported

Name:

Define comparative groups Numerator:	Denominator:	
☐ HR ☐ OR ☐ RR		☐ not reported
☐ 95% CI		☐ not reported
If HR/OR not reported, was the association statistically significant?	☐Yes ☐ No	☐ not reported

Name:

Define comparative groups Numerator:	Denominator:	
☐ HR ☐ OR ☐ RR		☐ not reported
☐ 95% CI		☐ not reported
If HR/OR not reported, was the association statistically significant?	☐Yes ☐ No	☐ not reported

Name:

Define comparative groups Numerator:	Denominator:	
☐ HR ☐ OR ☐ RR		☐ not reported
☐ 95% CI		☐ not reported
If HR/OR not reported, was the association statistically significant?	☐Yes ☐ No	☐ not reported

Continuous variables

Name:

Define comparative groups	Numerator:	Denominator:	
☐ HR ☐ OR ☐ RR			☐ not reported
☐ 95% CI			☐ not reported
If HR/OR not reported, was the association statistically significant?		☐Yes ☐ No	☐ not reported

Name:

Define comparative groups	Numerator:	Denominator:	
☐ HR ☐ OR ☐ RR			☐ not reported
☐ 95% CI			☐ not reported
If HR/OR not reported, was the association statistically significant?		☐Yes ☐ No	☐ not reported

Name:

Define comparative groups	Numerator:	Denominator:	
☐ HR ☐ OR ☐ RR			☐ not reported
☐ 95% CI			☐ not reported
If HR/OR not reported, was the association statistically significant?		☐Yes ☐ No	☐ not reported

Name:

Define comparative groups	Numerator:	Denominator:	
☐ HR ☐ OR ☐ RR			☐ not reported
☐ 95% CI			☐ not reported
If HR/OR not reported, was the association statistically significant?		☐Yes ☐ No	☐ not reported

Name:

Define comparative groups	Numerator:	Denominator:	
☐ HR ☐ OR ☐ RR			☐ not reported
☐ 95% CI			☐ not reported
If HR/OR not reported, was the association statistically significant?		☐Yes ☐ No	☐ not reported

Name:

Define comparative groups	Numerator:	Denominator:	
☐ HR ☐ OR ☐ RR			☐ not reported
☐ 95% CI			☐ not reported
If HR/OR not reported, was the association statistically significant?		☐Yes ☐ No	☐ not reported

Name:

Define comparative groups	Numerator:	Denominator:	
☐ HR ☐ OR ☐ RR			☐ not reported
☐ 95% CI			☐ not reported
If HR/OR not reported, was the association statistically significant?		☐Yes ☐ No	☐ not reported

Name:

Define comparative groups	Numerator:	Denominator:	
☐ HR ☐ OR ☐ RR			☐ not reported
☐ 95% CI			☐ not reported
If HR/OR not reported, was the association statistically significant?		☐Yes ☐ No	☐ not reported

Name:

Define comparative groups	Numerator:	Denominator:	
☐ HR ☐ OR ☐ RR			☐ not reported
☐ 95% CI			☐ not reported
If HR/OR not reported, was the association statistically significant?		☐Yes ☐ No	☐ not reported

Name:

Define comparative groups	Numerator:	Denominator:	
☐ HR ☐ OR ☐ RR			☐ not reported
☐ 95% CI			☐ not reported
If HR/OR not reported, was the association statistically significant?		☐Yes ☐ No	☐ not reported

(E) ASSESSMENT OF STUDY QUALITY

Consecutive or random patient selection	☐Yes	☐ No	☐ Not mentioned
Selection of predictors	☐ Clinical importance	☐ Statistical significance	☐ Not mentioned
Missing values	☐ No | ☐ Imputation	☐ Exclusion	☐ Not mentioned
Percentage of patients lost to follow-up			☐ Not mentioned
Linearity for continuous predictors	☐Yes | ☐ No	☐ Not applicable	☐ Not mentioned
Over-fitting (ratio events/predictor <10). Specify for which outcome, if more than one outcome evaluated	☐Yes	☐ No	☐ Not mentioned
Model assumptions held (i.e. proportional hazards)	☐Yes	☐ No	☐ Not mentioned
External or Internal validation (bootstrapping)	☐Yes	☐ No	☐ Not mentioned

(F) QUIPS

Refer to QUIPS guidelines to assess quality of study
