# Supplementary material for: Risk Estimation of Severe Primary Graft Dysfunction in Heart Transplant Recipients Using a Smartphone
Source: Rev Cardiovasc Med. 2025 Jan 8;26(1):25170. doi: 10.31083/RCM25170 (PMC11759961; doi:10.31083/RCM25170)
Supplement: Supplementary file 1 [file 2153-8174-26-1-25170-s1.zip › Supplementary C Quality assessment of individual studies.pdf]

# Appendix C summarizes the quality assessment of individual studies

| First author | year | Study participants | Prognostic factors measurement | Outcome measurement | Study confounding | Statistical analysis & reporting | Overall risk of bias |
|---|---|---|---|---|---|---|---|
| **Buchan TA** | 2021 | | Moderate | Low | Moderate | Low | Low |
| **Benck L** | 2021 | Low | Low | Low | Moderate | Low | Low |
| **King PM** | 2020 | Low | Low | Low | Low | Low | Low |
| **Truby LK** | 2018 | Moderate | Low | Low | High | Low | Moderate |
| **Nicoara A** | 2018 | Low | Low | Low | Low | Low | Low |
| **Younju R** | 2021 | Low | Low | Low | Low | Low | Low |
