# Supplementary material for: Risk Estimation of Severe Primary Graft Dysfunction in Heart Transplant Recipients Using a Smartphone
Source: Rev Cardiovasc Med. 2025 Jan 8;26(1):25170. doi: 10.31083/RCM25170 (PMC11759961; doi:10.31083/RCM25170)
Supplement: Supplementary file 1 [file 2153-8174-26-1-25170-s1.zip › Supplementary D sub group analysis for outcome.pdf]

Appendix D Sub-group analysis for outcome

Donor age > 40 years old

Study	Log (HR)	SE	Weight	HR 95% CI
Buchan TA 2021	0.4637	0.7878	33.8%	1.75 (0.70, 3.90)
Benck L 2021	0.0995	0.2062	16.7%	1.10 (0.74, 1.65)
King PM 2020	0.1897	0.2094	13.9%	1.84 (0.80, 1,.9)
Truby LK 2018	0.0497	0.5084	9.9%	1.04 (0.60, 1.06)
Nicoara A 2018	0.5801	0.4076	13.9%	1.08 (0.88, 1.03)
Younju R 2021	0.0877	0.2377	9.4%	1.02 (1.00, 1.03)
**Total (95%CI)**			**100.0%**	**1.65 (0.80, 3.40)**

Heterogeneity $Tau^2 = 0.00$; $Chi^2 = 9.18$; $I^2 = 57\%$
Test for overall effect: Z = 10.56 (p < 0.00001)

Donor female gender

Study	Log (HR)	SE	Weight	HR 95% CI
Buchan TA 2021	0.267	0.7878	39.1%	2.05 (0.72, 2.90)
Benck L 2021	0.0832	0.2062	15.7%	1.60 (0.94, 1.89)
King PM 2020	0.289	0.2094	11.9%	1.74 (0.60, 1.86)
Truby LK 2018	0.0497	0.5084	10.9%	1.04 (0.69, 1.32)
Nicoara A 2018	0.801	0.4076	11.7%	1.08 (0.78, 1.03)
Younju R 2021	0.0867	0.1375	10.7%	1.94 (1.50, 2.43)
**Total (95%CI)**			**100.0%**	**2.07 (1.21, 3.25)**

Heterogeneity $Tau^2 = 0.01$; $Chi^2 = 2.23$; $I^2 = 49\%$
Test for overall effect: Z = 8.26 (p < 0.00001)

Gender mismatch

Study	Log (HR)	SE	Weight	HR 95% CI
Buchan TA 2021	0.4127	0.3978	45.1%	2.55 (0.72, 2.88)
Benck L 2021	0.1895	0.0267	12.7%	1.70 (0.99, 1.78)
King PM 2020	0.1875	0.8090	11.4%	1.64 (0.69, 1.96)
Truby LK 2018	0.01	0.673	9.9%	1.34 (0.79, 1.39)
Nicoara A 2018	0.8790	0.9480	10.7%	1.88 (0.98, 2.32)
Younju R 2021	0.0986	0.147	10.2%	1.64 (1.50, 2.53)
**Total (95%CI)**			**100.0%**	**2.43 (1.10, 5.38)**

Heterogeneity $Tau^2 = 0.09$; $Chi^2 = 4.23$; $I^2 = 59\%$
Test for overall effect: Z = 1.86 (p < 0.00001)

Undersized donor

Study	Log (HR)	SE	Weight	HR 95% CI
Buchan TA 2021	0.212	0.397	45.1%	3.55 (1.92, 4.28)
Benck L 2021	0.095	0.2067	12.7%	1.70 (1.39, 2.00)
King PM 2020	0.1875	0.090	12.4%	1.84 (0.79, 1.99)
Truby LK 2018	0.1235	0.2673	10.9%	1.34 (0.79, 1.39)
Nicoara A 2018	0.01	0.1	13.7%	2.88 (1.98, 3.00)
Younju R 2021	0.157	0.180	5.2%	1.64 (1.30, 2.03)
**Total (95%CI)**			**100.0%**	**3.33 (1.61, 6.67)**

Heterogeneity $Tau^2 = 0.00$; $Chi^2 = 34.23$; $I^2 = 55\%$
Test for overall effect: Z = 7.78 (p < 0.00001)

Pre operatory LVAD support

Study	Log (HR)	SE	Weight	HR 95% CI
Buchan TA 2021	0.778	0.650	65.1%	2.55 (1.62, 3.28)
Benck L 2021	0.195	0.20	6.7%	1.88 (1.59, 2.10)
King PM 2020	0.012	0.50	8.4%	1.09 (0.79, 1.39)
Truby LK 2018	0.389	0.890	5.9%	1.24 (0.99, 1.36)
Nicoara A 2018	0.023	0.4	10.7%	2.66 (1.68, 3.00)
Younju R 2021	0.150	0.038	3.2%	1.74 (1.80, 2.03)
**Total (95%CI)**			**100.0%**	**2.40 (1.02, 5.64)**

Heterogeneity $Tau^2 = 0.00$; $Chi^2 = 54.23$; $I^2 = 65\%$
Test for overall effect: Z = 13.08 (p < 0.00001)

Recipient Amiodarone treatment

Study	Log (HR)	SE	Weight	HR 95% CI
Buchan TA 2021	0.998	0.250	69.9%	2.25 (1.92, 2.98)
Benck L 2021				
King PM 2020	0.012	0.980	18.4%	3.91 (0.79, 1.39)
Truby LK 2018	0.089	0.490	5.8%	1.01 (0.69, 1.16)
Nicoara A 2018				
Younju R 2021				

**Total (95%CI)**			**100.0%**	**2.14 (1.39, 3.29)**

Heterogeneity $Tau^2 = 0.01$; $Chi^2 = 54.23$; $I^2 = 50\%$
Test for overall effect: Z = 11.09 ($p < 0.00001$)

Recipient Diabetes

Study	Log (HR)	SE	Weight	HR 95% CI
Buchan TA 2021	0.778	0.057	70.1%	2.55 (1.62, 3.28)
Benck L 2021	0.139	0.229	16.7%	2.75 (1.59, 2.90)
King PM 2020				
Truby LK 2018	0.08	0.090	5.9%	1.24 (0.99, 1.36)
Nicoara A 2018				
Younju R 2021	0.01	0.038	7.3%	2.22 (1.42, 3.46)
**Total (95%CI)**			**100.0%**	**3.04 (1.39, 3.29)**

Heterogeneity $Tau^2 = 0.01$; $Chi^2 = 5.23$; $I^2 = 45\%$
Test for overall effect: Z = 3.08 ($p < 0.00001$)

Recipient Creatinine level > 250mg/dl

Study	Log (HR)	SE	Weight	HR 95% CI
Buchan TA 2021	0.748	0.257	73.9%	3.26 (2.18, 6.71)
Benck L 2021	0.489	0.229	12.7%	2.95 (1.59, 4.98)
King PM 2020				
Truby LK 2018	0.658	0.190	13.4%	3.84 (2.99, 4.36)
Nicoara A 2018				
Younju R 2021				
**Total (95%CI)**			**100.0%**	**3.66 (2.08, 6.51)**

Heterogeneity $Tau^2 = 0.00$; $Chi^2 = 8.23$; $I^2 = 55\%$
Test for overall effect: Z = 12.08 ($p < 0.00001$)

Graft ischemic time > 180 min

Study	Log (HR)	SE	Weight	HR 95% CI
Buchan TA 2021	0.108	0.457	43.9%	1.01 (1.01, 1.01)
Benck L 2021	0.089	0.029	22.7%	2.95 (1.09, 4.08)
King PM 2020				
Truby LK 2018	0.800	0.090	23.4%	2.04 (2.00, 2.36)
Nicoara A 2018				
Younju R 2021	0.799	0.129	10%	1.79 (1.06, 3.28)
**Total (95%CI)**			**100.0%**	**2.15 (1.81, 2.68)**

Heterogeneity $Tau^2 = 0.01$; $Chi^2 = 6.33$; $I^2 = 35\%$
Test for overall effect: Z = 2.98 ($p < 0.00001$)

By-pass time > 120 min

Study	Log (HR)	SE	Weight	HR 95% CI
Buchan TA 2021				
Benck L 2021	0.089	0.720	42.7%	2.95 (1.59, 4.98)
King PM 2020				
Truby LK 2018	0.601	0.090	57.3%	1.84 (2.09, 3.36)
Nicoara A 2018				
Younju R 2021				
**Total (95%CI)**			**100.0%**	**2.53 (2.00, 3.89)**

Heterogeneity $Tau^2 = 0.00$; $Chi^2 = 1.23$; $I^2 = 44\%$
Test for overall effect: Z = 1.87 ($p < 0.00001$)

Previous sternotomy

Study	Log (HR)	SE	Weight	HR 95% CI
Buchan TA 2021	0.748	0.257	63.6%	3.26 (2.18, 6.71)
Benck L 2021	0.489	0.229	5.7%	2.95 (1.59, 4.98)
King PM 2020	0.099	0.080		2.11 (1.08, 2;98)
Truby LK 2018	0.649	0.720	23.4%	2.84 (2.99, 3.36)
Nicoara A 2018	0.016	0.010	7.3%	2.99 (1.98, 5,23)
Younju R 2021				
**Total (95%CI)**			**100.0%**	**3.21 (1.32, 7.80)**

Heterogeneity $Tau^2 = 0.01$; $Chi^2 = 1.63$; $I^2 = 61\%$
Test for overall effect: Z = 1.00 ($p < 0.00001$)
